# Discovery and Genome Annotation of Actinobacteriophages RazzB and SwissCheezer Isolated From Soil in Southwestern Pennsylvania

**DOI:** 10.17912/micropub.biology.001995

**Published:** 2026-03-10

**Authors:** Christina H. Zagorac, Jacky Abbdoush, Abraham X. Chieng, Madison L. Cottle, Ashley N. Giles, Zoey E. Hughes, Rebecca L. Huss, Kaprice Johnson, David Lachance, Jeramiah L. McDermott, Paige A. Phillips, Ryan Ritson, Jenna Sonnie, Tristan H. Todd, Jessica K. Wallace, Julia E. Wilson, Sarah J. Swerdlow, Jennifer R. Ingram

**Affiliations:** 1 Biology, University of Pittsburgh at Greensburg

## Abstract

Bacteriophages RazzB and SwissCheezer were isolated from soil in southwestern Pennsylvania. RazzB is a cluster AP siphovirus infecting&nbsp; *Arthrobacter globiformis* B-2979 and contains a 69,522 bp genome with 65.8% GC content, 127 predicted protein-coding genes and no tRNAs. SwissCheezer is a cluster EK phage with a podovirus morphology infecting *Microbacterium foliorum* NRRL B-24224 and contains a 53,956 bp genome with 60.0% GC content and 54 predicted protein-coding genes.

**Figure 1. Transmission electron micrographs of RazzB (A) and SwissCheezer (B) reveal two distinct morphologies:**
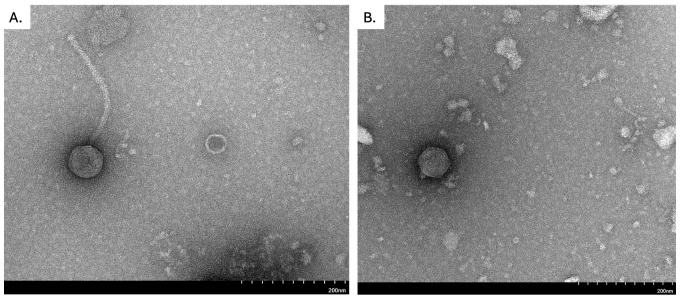
Scale bar is 200 nm.

## Description

Soil is a rich source for isolating diverse and novel bacteriophages, the characterization of which contributes towards advancing our understanding of phage genetic diversity and application of phages in medicine and biotechnology (Hatfull, 2022; Pope et al., 2015). Here, we present the genome sequences of newly discovered actinobacteriophages RazzB and SwissCheezer. Bacteriophages RazzB and SwissCheezer were discovered in regions of southwestern Pennsylvania. RazzB was discovered via enriched isolation on *Arthrobacter globiformis B-2979* from soil located near a raspberry bush in a family garden in Mifflintown, PA, USA (GPS: 40.53858 N, 77.69635 W), while SwissCheezer was discovered by direct isolation on *Microbacterium foliorum NRRL B-24224* from soil at a residential home in Greensburg, PA, USA (GPS: 40.30196 N, 79.53702 W). The sample containing RazzB was suspended in 35 mL of peptone yeast calcium (PYCa) medium at 30°C for 45 minutes, centrifuged at 2,000 x g for 10 minutes, and filtered using a 0.22 µm syringe. After inoculation of the filtrate with *Arthrobacter globiformis B-2979* and incubation at 30°C for 48 hours, the resulting culture was filtered and the filtrate spotted on a bacterial lawn of *A. globiformis* (Poxleitner et. al, 2024). For SwissCheezer, the filtrate prepared after resuspending the soil sample was plated directly onto PYCa top agar with *Microbacterium foliorum NRRL B-2422* 4 and incubated for 24 hours at 30°C. Both phages were purified through a total of three rounds of picking individual plaques and plating. RazzB formed small, clear plaques between 1.3 -1.5 mm in diameter (n=5) whereas SwissCheezer formed clear plaques that consistently ranged in size, from 1 – 4 mm in diameter (n = 5). Lysates for both phages were prepared and used for negative-staining transmission electron microscopy with 1% uranyl acetate, revealing a siphovirus morphology for RazzB, with a tail length mean of 236nm (+/-8 nm) and a head diameter mean of 68 nm (+/- 4 nm) (n=3) and a podovirus morphology for SwissCheezer, with a mean head diameter of 66nm (+/- 8 nm) (n=3) (Figure 1).

DNA was extracted from phage lysates using Promega Wizard DNA Clean-Up Kit. Sequencing for these genomes was completed by the Pittsburgh Bacteriophage Institute using an Illumina NextSeq 1000 using the XLEAP P1 kit (Russell and Hatfull, 2017) generating 3.1 million 100 base reads. Raw reads were trimmed with cutadapt 4.7 (using the option: –nextseq-trim 30) and filtered with skewer 0.2.2 (using the options: -q 20 -Q 30 -n -l 50) prior to assembly (Gordon et al., 1998; Jiang et al., 2014; Martin, 2011; Wick et al., 2017). A summary of the DNA sequencing and genome characteristics of RazzB and SwissCheezer is found in Table 1. RazzB was assigned to cluster AP and SwissCheezer to cluster EK based on gene content similarity of at least 35% to phages in the Actinobacteriophage database, phagesdb (https://phageDB.org) (Pope et al., 2017; Russell and Hatfull, 2017).

The genomes of RazzB and SwissCheezer were auto-annotated using GLIMMER v3.02 (Delcher et al., 1999) and GeneMark v2.5 (Besemer & Borodovsky, 2005) and then manually refined with Phamerator using the Actino_draft database v580 (Cresawn et al., 2011), DNA Master v5.23.6 (Pope and Jacobs-Sera, 2018), Starterator v587 (github.com/SEA-PHAGES/starterator), and PECAAN (discover.kbrinsgd.org). Functions were assigned to predicted genes in RazzB and SwissCheezer genomes by using BLASTp v 2.2.26 (using the Actinobacteriophage and NCBI nonredundant databases; Altschul et al., 1990) and HHpred (using PDB_mmCIF70, Pfam-v.36, and NCBI Conserved Domain databases; Söding et al., 2005) as well as determining transmembrane regions using Deep TMHMM v1.0.42 (Hallgren et al., 2022) program. No tRNA genes were identified by Aragorn v 1.2.38 (Laslett & Canback, 2004) or tRNAscan-SE v2.0 (Lowe & Chan, 2016) in either phage. All programs were run using default settings except for DNA Master, which was set as outlined in the SEA-PHAGES bioinformatics guide (Pope et al., 2017).

Of the 127 predicted protein-coding genes in RazzB's genome, genes 1-45 are transcribed in the forward direction and genes 46-127 in the reverse direction. Structural and assembly genes are located in the first third of the genome and include those encoding the terminase, portal protein, capsid maturation protease, major capsid hexamer protein, tail tube, head-to-tail adapter, tail-assembly chaperone, tape measure protein, distal tip complex (dit), baseplate hub, and minor tail proteins. Genes involved in replication and recombination, such as a RusA-like resolvase and a DNA primase/helicase, were also identified. The endolysin gene is positioned upstream of the tape measure gene, consistent with other subcluster AP2 phages. RazzB encodes a Cas4 exonuclease, dUTPase, and a protein containing a ParB-like nuclease domain that are conserved among AP2 cluster phages.

SwissCheezer contains 54 predicted protein-coding genes, with genes 1-30 transcribed in the reverse direction and genes 31-54 in the forward direction, a layout typical for subcluster EK1 phages. Genes involved in replication and recombination like DNA primase/polymerase, DNA primase, DNA helicase, DNA polymerase I, and a RuvC-like resolvase are encoded in the first part of the genome. Structural genes such as portal protein, major capsid protein, and minor tail proteins are encoded in the last third of the genome along with an endolysin on the forward strand. Functions fully conserved across all 57 EK1 phages include minor tail proteins, endolysin, a WhiB family transcription factor and a Cas4 exonuclease. A notable variation amongst EK1 phages is within the rightmost fifth of the genome, where phages either encode purple acid phosphatase, as does SwissCheezer, a phosphoesterase, or are missing this coding region.

GenBank with Accession and Sequence Read Archive (SRA) numbers are provided in Table 1.

Table 1. Genome and sequencing characteristics of phages RazzB and SwissCheezer.

**Table:** 

Genome Characteristic	RazzB	SwissCheezer
Genome length (bp)	69,522	53,956
Sequencing coverage (X)	4,392	77
Character of genome ends	Direct terminal repeats (587 bp)	Circularly permuted
Number of predicted protein-coding genes	127	54
GC content (%)	65.8%	60.0%
Subcluster	AP2	EK1
GenBank Accession Number	PV876951	PV876939
SRA Accession Number	SRX31241828	SRX31241830

## References

[R1] Altschul Stephen F., Gish Warren, Miller Webb, Myers Eugene W., Lipman David J. (1990). Basic local alignment search tool. Journal of Molecular Biology.

[R2] Besemer J., Borodovsky M. (2005). GeneMark: web software for gene finding in prokaryotes, eukaryotes and viruses. Nucleic Acids Research.

[R3] Cresawn Steven G, Bogel Matt, Day Nathan, Jacobs-Sera Deborah, Hendrix Roger W, Hatfull Graham F (2011). Phamerator: a bioinformatic tool for comparative bacteriophage genomics. BMC Bioinformatics.

[R4] Delcher Arthur L., Bratke Kirsten A., Powers Edwin C., Salzberg Steven L. (2007). Identifying bacterial genes and endosymbiont DNA with Glimmer. Bioinformatics.

[R5] Gordon David, Abajian Chris, Green Phil (1998). *Consed:*
A Graphical Tool for Sequence Finishing. Genome Research.

[R6] Hallgren Jeppe, Tsirigos Konstantinos D., Pedersen Mads Damgaard, Almagro Armenteros José Juan, Marcatili Paolo, Nielsen Henrik, Krogh Anders, Winther Ole (2022). DeepTMHMM predicts alpha and beta transmembrane proteins using deep neural networks.

[R7] Hatfull Graham F. (2022). Mycobacteriophages: From Petri dish to patient. PLOS Pathogens.

[R8] Jiang Hongshan, Lei Rong, Ding Shou-Wei, Zhu Shuifang (2014). Skewer: a fast and accurate adapter trimmer for next-generation sequencing paired-end reads. BMC Bioinformatics.

[R9] Laslett D. (2004). ARAGORN, a program to detect tRNA genes and tmRNA genes in nucleotide sequences. Nucleic Acids Research.

[R10] Lowe Todd M., Chan Patricia P. (2016). tRNAscan-SE On-line: integrating search and context for analysis of transfer RNA genes. Nucleic Acids Research.

[R11] Martin Marcel (2011). Cutadapt removes adapter sequences from high-throughput sequencing reads. EMBnet.journal.

[R12] Pope Welkin H, Bowman Charles A, Russell Daniel A, Jacobs-Sera Deborah, Asai David J, Cresawn Steven G, Jacobs William R, Hendrix Roger W, Lawrence Jeffrey G, Hatfull Graham F, Science Education Alliance Phage Hunters Advancing Genomics and Evolutionary Science, Phage Hunters Integrating Research and Education, Mycobacterial Genetics Course (2015). Whole genome comparison of a large collection of mycobacteriophages reveals a continuum of phage genetic diversity. eLife.

[R13] Pope Welkin H., Mavrich Travis N., Garlena Rebecca A., Guerrero-Bustamante Carlos A., Jacobs-Sera Deborah, Montgomery Matthew T., Russell Daniel A., Warner Marcie H., Hatfull Graham F., Science Education Alliance-Phage Hunters Advancing Genomics and Evolutionary Science (SEA-PHAGES) (2017). Bacteriophages of
*Gordonia*
spp. Display a Spectrum of Diversity and Genetic Relationships. mBio.

[R14] Pope WH, Jacobs-Sera D (2018). Annotation of Bacteriophage Genome Sequences Using DNA Master: An Overview.. Methods Mol Biol.

[R15] Pope WH, Jacobs-Sera DJ, Russell DA, Cresawn SG, Hatfull GF. 2017a. HHMI SEA-PHAGES Bioinformatics Guide. https://seaphagesbioinformatics.helpdocsonline.com/home. Accessed 1 December 2025.

[R16] Poxleitner M, Pope WH, Jacobs-Sera D, Sivanathan V, Hatfull GF. 2024. *Phage discovery guide* . Howard Hughes Medical Institute, Chevy Chase, MD. Available from: https://seaphagesphagediscoveryguide.helpdocsonline.com/5-6-protocol. Accessed 17 February 2026.

[R17] Rinehart CA, Gaffney B, Wood, JD, Smith J. 2016. PECAAN, a Phage Evidence Collection and Annotation Network.https://discover.kbrinsgd.org/login. Accessed 1 December 2025.

[R18] Russell Daniel A, Hatfull Graham F (2016). PhagesDB: the actinobacteriophage database. Bioinformatics.

[R19] Soding J., Biegert A., Lupas A. N. (2005). The HHpred interactive server for protein homology detection and structure prediction. Nucleic Acids Research.

[R20] Wick Ryan R., Judd Louise M., Gorrie Claire L., Holt Kathryn E. (2017). Unicycler: Resolving bacterial genome assemblies from short and long sequencing reads. PLOS Computational Biology.

